# Plasticity of Epigenomic and Transcriptomic Aging Reveals Common Targets for Reprogramming by Environmental Exposures

**DOI:** 10.21203/rs.3.rs-7368222/v1

**Published:** 2025-09-19

**Authors:** Cheryl Walker, Sandra Grimm, Rahul Jangid, Marisa Bartolomei, Dana Dolinoy, David Aylor, Gokhan Mutlu, Shyam Biswal, Bo Zhang, Robert Hamanaka, Justin Colacino, Maureen Sartor, Laurie Svoboda, Ting Wang, Cristian Coarfa

**Affiliations:** Baylor College of Medicine; Baylor College of Medicine; Baylor College of Medicine; University of Pennsylvania; University of Michigan School of Public Health; North Carolina State University; University of Chicago; Johns Hopkins University; Washington University School of Med; The University of Chicago; University of Michigan School of Public Health; University of Michigan School of Public Health; University of Michigan; Washington University in St. Louis; Baylor College of Medicine

## Abstract

Environmental exposures during early life are increasingly recognized as key determinants of health and disease in adulthood (1) but how they durably shape disease risk across the lifecourse remains poorly understood. Here, we show early-life toxicants reprogram the epigenome and redirect age-associated transcriptional trajectories—polarizing cell-specific gene expression and predisposing to liver disease. The TaRGET II Consortium (2) exposed mice to diverse toxicants from pre-conception through weaning and followed individual animals though adulthood with multi-omic profiling. Analysis of >800 liver epigenomic and transcriptomic profiles from male and female mice revealed that despite differing chemical classes and mechanisms of action, multiple toxicants — BPA, TBT, TCDD, and PM2.5 — produced exposure signatures that converged on genes normally differentially expressed in the liver as animals aged. Histone modifications at enhancers emerged as key targets for epigenomic reprogramming of these liver aging-associated plasticity genes (LAAsP genes). Reprogrammed LAAsP genes exhibited a striking, bidirectional signature. In hepatocytes LAAsP genes that typically increase with age, such as those involved in metabolism, were repressed. Conversely, in non-parenchymal cells, LAAsP genes that normally decline with age, including those for extracellular matrix production, remained elevated. An attenuated LAAsP gene signature and polarized transcriptional states were mirrored in human liver disease and hepatocellular carcinoma and could effectively distinguished healthy from diseased human liver transcriptomes. Together, these findings demonstrate that early-life environmental exposures can hijack the plasticity of epigenomic aging, durably reprogram expression trajectories, and lock in polarized states that foreshadow chronic liver disease and cancer.

## Introduction

Epigenetic programming, once established, is heritable, yet retains the intrinsic plasticity necessary for three fundamental biological processes: remodeling the epigenome for cell-type specification during development, resetting imprinted loci for parent-of-origin-specific allele expression, and programmed epigenomic aging that directs changes in gene expression across the life course ^3,4^. Beyond these essential biologies, the epigenome is also susceptible to reprogramming by environmental exposures, particularly during critical windows of development ^5^. Both human epidemiological and animal model studies have demonstrated that environmental factors—including nutritional status, stress, and toxicant exposure—can induce lasting changes in the epigenome, with effects that persist long after the exposure has ceased.

Targets for epigenetic reprogramming include DNA methylation, histone modifications, and non-coding RNAs. Importantly, perturbation of the epigenome can lock in abnormal gene expression patterns, contributing to disease susceptibility in later life ^6,7^. This persistent epigenetic reprogramming is implicated in a wide range of adverse health outcomes, including metabolic disorders, cardiovascular diseases, and cancer ^7,8^. In some instances, these changes may even be transmitted to subsequent generations, compounding the long-term effects of the initial exposure through transgenerational epigenetic inheritance ^9,10^.

The Toxicant Exposures and Responses by Genomic and Epigenomic Regulators of Transcription II (TaRGET II) consortium (T2C) was established by the National Institute of Environmental Health Sciences (NIEHS) to understand how the epigenome and transcriptome respond to early-life environmental exposures and evaluate epigenomic signatures as biomarkers of exposure and predictors of disease risk ^2^. TaRGET II investigated a wide array of environmental toxicants, with diverse mechanisms of action. These included ligand activation of nuclear hormone receptors—such as the estrogen receptor, peroxisome proliferator-activated receptor, and aryl hydrocarbon receptor—triggered by compounds bisphenol A (BPA), tributyltin (TBT), and dioxin (TCDD) ^11,12^. The consortium also explored toxicants inducing oxidative stress, such as particulate matter (PM2.5) ^13^, agents that disrupt normal enzymatic, ion channel, and receptor function, such as lead (Pb) ^14^, and the endocrine disrupting chemical (EDC) Di(2-ethylhexyl) phthalate (DEHP) ^15^. All exposures began pre-conception and continued through weaning, after which exposure ceased, and animals were followed longitudinally into early (5 months) and later (10 months) adulthood to assess lasting effects. Further details on the exposures and methodologies employed across the consortium can be found in the Materials and Methods and publicly accessible metadata through the TaRGET II data portal (https://dcc.targetepigenomics.org/) and an accompanying database (http://toxitarget.com/).

In mice, the liver undergoes distinct morphological and functional transitions during embryonic and perinatal development, with well-characterized transcriptional correlates for this period of liver maturation ^16^. Significant transcriptional changes also occur in very old animals- particularly between 12 to 24 months of life- as highlighted by recent comprehensive studies ^17,18^. However, less is known about the epigenomic and transcriptomic shifts occurring between weaning, early and later adulthood—the interval selected by the T2C to assess the persistent effects of early-life environmental exposures. Therefore, to lay a foundation for the exposure studies, longitudinal profiling of normal livers was conducted from 3 weeks to 10 months, examining histone modifications (this study) and DNA methylation ^19^,as well as transcriptomic changes via RNA sequencing (RNA-seq). The eight exposure groups were two doses of BPA: 10 mg/kg-diet/day (BPA.hi) and 10 μg/kg-diet/day (BPA.lo) DEHP (5mg/kg-diet/day), Pb (32 ppm-drinking water), TBT (3 uM-drinking water), TCDD (1 ug/kg-diet/day), and PM2.5 exposures conducted by two different consortium sites—Johns Hopkins University (PM2.5-JHU, concentrated PM2.5: 150 μg/m^3^) and the University of Chicago (PM2.5-CHI, synthetic PM2.5: 150 μg/m^3^) (Supplemental Figure 1a). From these study animals, the Consortium profiled individual mice, and for the present analysis utilized 410 transcriptomes (RNA-seq for males and females) and 436 epigenomes (ChIP-seq of males) for the eight different exposure groups and sex- and age-matched vehicle controls, at 3 different ages (weaning at 3 weeks, early adulthood at 5 months and later adulthood at 10 months), all without pooling samples. This individualized deep-sequencing approach enabled detection of exposure-specific transcriptional and epigenomic reprogramming while preserving the ability to account for inter-individual biological variability in response.

## RESULTS

### Early-Life Exposures Cause Persistent Disruption of the Adult Liver Transcriptome

**Figure 1:**
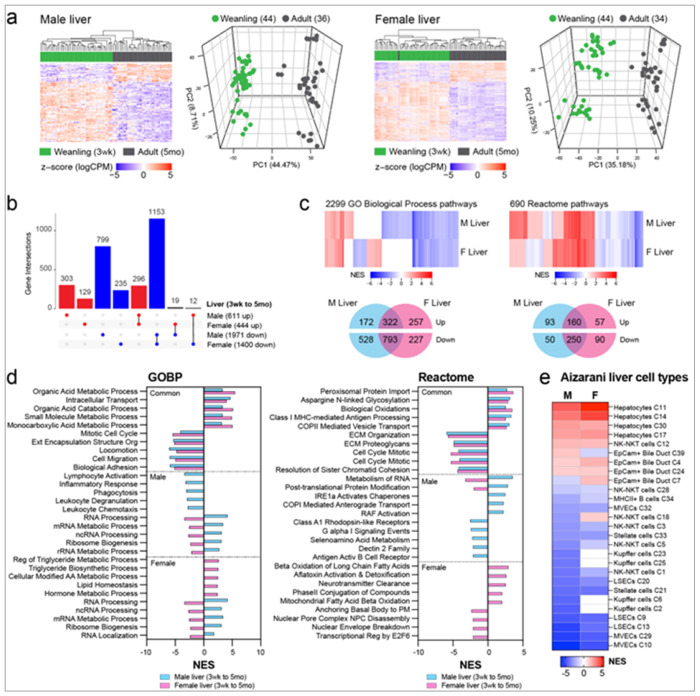
Transcriptomic aging signatures for male and female mice a. PCA and heat maps of transcriptional changes associated with aging from weanling (3wk) to adult (5mo). b. UpSet plot comparing the aging transcriptome in males and females c. GSEA enriched pathways in male and female based on Gene Ontology Biological Processes (GOBP) and Reactome compendia. d. Top shared and sex-specific GOBP and Reactome enriched pathways changed with age. e. GSEA enrichment results for liver cell type markers in male and female mice.

As the mouse liver ages from weaning (3 weeks) to early adulthood (5 months), many genes exhibit differential expression, as demonstrated by the principal component analysis (PCA) and heatmaps presented in Figure 1a, along with UpSet plots in Figure 1b, which show numbers of male-specific, female-specific and shared (i.e. occurring in both sexes) gene expression changes. In both sexes, the majority of differentially expressed genes (DEGs, FDR <0.05, fold change exceeding 1.5x) that changed as a function of age exhibited a decrease in expression during this period: 1,153 DEGs were significantly downregulated in young adulthood in both males and females, compared to 296 DEGs that increased significantly during this time (Figure 1b). Furthermore, the majority of these DEGs exhibited sex-specificity: we found 799 male-specific genes decreased in expression versus 303 that increased, and 235 female-specific genes that decreased compared to 129 that increased. Table 1 highlights the top DEGs upregulated and downregulated in males and females with age, with the complete list of all DEGs available in the combined signature file in Supplemental Table 1. Together, this analysis identified 2582 genes in males and 1844 genes in females that retained the plasticity to significantly change expression as animals aged, which here are termed liver aging–associated plasticity (LAAsP) genes.

Gene set enrichment analysis (GSEA) revealed many functional pathways that were positively or negatively enriched for LAAsP genes in both sexes. These are shown in Figure 1c–d, and Suppl Figure 1a-b; Supplemental Table 2 provides a complete list of enriched pathways and their core genes. Employing GO-BP and Reactome (Figure 1c–d) and Hallmark and KEGG compendia (Supplemental Figure 1b-c), pathways with the greatest enrichment in both males and females were associated with extracellular matrix production and cytoskeletal functions such as locomotion and mitosis (most negatively enriched) and cellular metabolism and peroxisome function (most positively enriched). Interestingly, some pathways, such as those related to RNA processing, exhibited opposing trends in males (upregulated) versus females (downregulated) (Figure 1c).

When GSEA was applied against cell identity genes for parenchymal (epithelial) and non-parenchymal liver cells, clear cell-type and directional specificity emerged. Between weaning and young adulthood, pathways enriched for parenchymal hepatocyte and bile duct epithelial cell identity genes were predominantly upregulated, whereas pathways enriched for cell identity genes of non-parenchymal cells, such as Stellate, Kupffer and endothelial cells, were downregulated (Figure 1e), consistent with the known metabolic, extracellular matrix production and immune signaling functions of these cell types.

We next assessed the impact of diverse early-life environmental exposures on the transcriptome by RNA-seq. Transcriptional profiling was performed on livers from individual male and female mice from the eight exposure groups. Each group of exposed animals were compared to age- and sex-matched vehicle control animals using a minimum of N=3 per sex/age, with the litter counted as the N, as detailed in Supplementary Materials and Methods. This profiling revealed exposure-specific, persistent disruption of the transcriptome, and identified vulnerabilities exploited by multiple T2C exposures.

**Figure 2:**
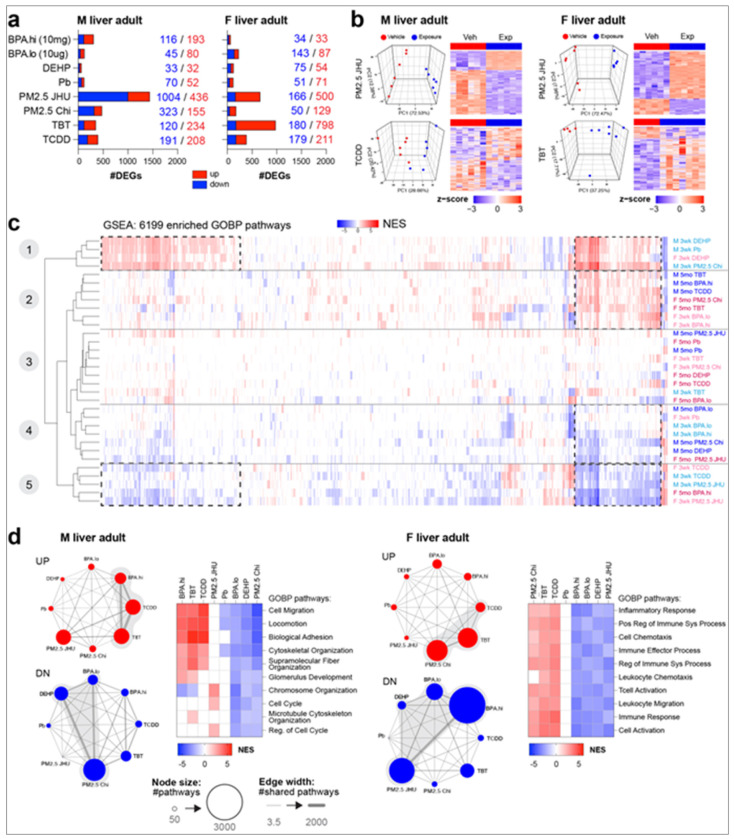
Impact of early-life toxicant exposures on the transcriptome. a. Summary of DEGs in male and female mice at 5 months for all 8 exposures compared to their respective vehicle controls. b. PCA and heatmaps for selected female and male mice exposures. c. Enriched pathways identified using GSEA against GOBP in male and female mouse livers for all 8 exposures. d. Network analysis of overlapping pathways in the same direction across the 8 exposures for male and female mice at 5 months. The node size is proportional to the number of enriched pathways; the edge thickness is proportional to the number of common pathways between two exposures. Overlaps indicated by grey shading are significant (p<10^−100^).

These findings are summarized for both sexes and all eight exposures in the bar graphs in Figure 2a, and all exposure DEGs are listed in the combined signature file in Supplemental Table 1. Representative PCA plots and heatmaps for males exposed to PM2.5-JHU and TCDD and females exposed to PM2.5-JHU and TBT are displayed in Figure 2b; comprehensive PCA plots, heatmaps, and volcano plots for all eight exposures and both sexes are provided in Supplemental Figure 2. While many early-life exposures induced persistent, long-lasting changes in the adult transcriptome, with numerous DEGs in their exposure signatures, this was not universally the case. The exposures eliciting the greatest to least disruption of the liver transcriptome in young adult male mice, as measured by number of DEGs, were PM2.5-JHU > PM2.5-CHI, TCDD, TBT, BPA.hi > BPA.lo, DEHP, Pb. In females, TBT > PM2.5-JHU, TCDD > BPA.lo, BPA.hi, DEHP, Pb, PM2.5-CHI (Figure 2a). There was also sex bias in how the transcriptome responded to several T2C exposures. For instance, as illustrated in Figure 2a, BPA.hi exposure induced >300 DEGs in livers of male mice but <70 DEGs in livers of exposed females, which were often littermates and therefore equivalently exposed.

To assess the potential for functional impact of exposure-induced disruptions of gene expression, we performed GSEA on the transcriptomes of weanling and young adult mice. As shown in Figure 2c (and Supplemental Table 2, which provides a complete list of enriched pathways and core genes), we found numerous pathways that were enriched, but remarkably, these pathways segregated into only 5 clusters. This is surprising given that this extensive data set was derived from mice at different ages (3 weeks and 5 months), both sexes, and in response to eight different exposures conducted at different consortium sites across the country. Subsequent analysis of the exposures and pathways that defined each of the 5 clusters offered valuable insights into how these early-life environmental exposures had shaped the transcriptome, and revealed common responses to multiple, diverse T2C exposures.

Clusters 1 and 5 were primarily defined by pathways enriched in weanlings (Figure 2c). Cluster 1 was characterized by pathways positively enriched in response to DEHP, Pb, and PM2.5-Chi, while Cluster 5 was defined by negative enrichment of these same pathways in response to TCDD, PM2.5-JHU, and BPA.hi. In contrast, Clusters 2 and 4 were characterized by pathways enriched in primarily young adult mice. Cluster 2 was characterized by pathways positively enriched in response to TBT, TCDD, BPA.hi, and PM2.5 and Cluster 4 by negative enrichment of these same pathways in response to BPA.lo, PM2.5, and DEHP. Notably, while many pathways were the same in weanlings and young adults, the exposures targeting them were different. As shown in the hatched boxes in Figure 2c, the exposures responsible for positive enrichment of the same pathways in weanlings and young adults in Clusters 1 and 2, respectively, were different, as were the exposures responsible for the negative enrichment of these same pathways in weanlings and young adults in Clusters 5 and 4, respectively. Thus, this surprising convergence revealed using unbiased clustering of pathways from different age-, sex-, and exposure-specific signatures, suggested there was a limited repertoire of pathways vulnerable to disruption, which became either activated or repressed depending on the specific exposure, age, and sex of the animals. This is illustrated in Suppl Figure 2b showing the top GOBP pathways positively enriched in Cluster 2 and negatively enriched in Cluster 4, which were pathways involved in cellular structure, adhesion and locomotion; pathways downregulated in Cluster 2 and upregulated in Cluster 4 were metabolic pathways, the most strongly enriched being RNA metabolism.

To investigate common targets for disruption by these early life exposures, we conducted network analyses to identify those pathways most vulnerable to disruption by T2C exposures—specifically, pathways enriched in response to multiple different exposures. The network diagrams in Figures 2d illustrate the number of enriched pathways (indicated by node size) and the overlaps between pathways enriched in response to the various exposures (represented by thick edges connecting the nodes). In young adult male livers, the nodes corresponding to BPA.hi, TCDD, and TBT (Cluster 2 exposures, Figure 2c, left) shared numerous positively enriched pathways, as indicated in the accompanying normalized enrichment score (NES) heatmap. These pathways included those related to cytoskeletal and fiber organization, adhesion, and locomotion (Figure 2d). Conversely, negatively enriched pathways exhibited strong overlaps between PM2.5-CHI, BPA.lo, and DEHP (Cluster 4 exposures), of these same pathways (Figure 2d). Similar trends were observed in adult female liver, as depicted in the network diagrams and heatmaps in Figure 2d, right. In female mice exposed to PM2.5-CHI, TBT, and TCDD, young adult livers displayed positive enrichment of pathways associated with immune response, inflammatory signaling, and cell migration, while conversely, livers from female mice exposed to BPA.hi, BPA.lo, DEHP, and PM2.5-JHU showed negative enrichment of these same pathways.

To confirm this striking finding that the signatures of diverse T2C exposed livers appeared to be enriched for many of the same pathways, we used permutation testing, which confirmed the significance of the shared pathways in the four networks from Figure 2d. This was followed by fitting a normal distribution and determining the z-score for all overlaps- those shown with gray shading in Figure 2d were highly significant (p<10^−100^). While the direction of enrichment (positive or negative) was exposure-specific, pathway enrichment also often exhibited sex specificity. The most significantly enriched pathways in exposed females involved processes for immune cell activation, while pathways related to adhesion and fiber organization were most enriched in the exposure signatures of males. This strong sex bias was not attributable to differential expression of X- or Y-linked genes: Expression of allosome-linked genes (108 X-linked and 64 Y-linked) accounted for <0.8% of the ~12,000 expressed protein coding genes in weanling or young adult livers. Furthermore, of the 5938 DEGs identified as LAAsP genes or changed in response to T2C exposures, 0 were Y-linked, and only 19 were X-linked (see Suppl Table 1b). A more detailed TaRGET II examination of how sex influenced responses to all exposures occurs in a forthcoming Consortium manuscript.

### Multiple Early-Life Exposures Disrupt the Aging Trajectory of Target Genes

**Figure 3:**
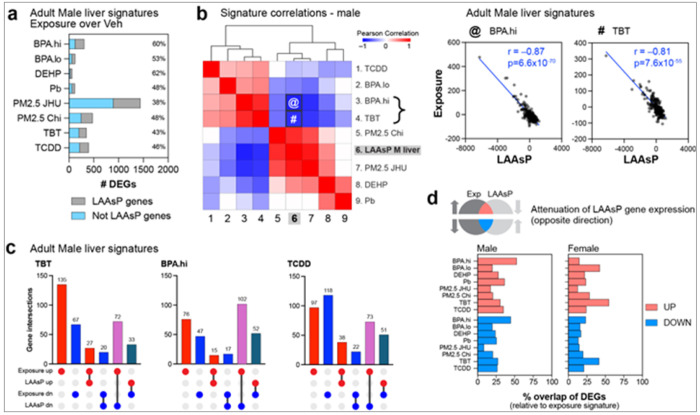
Robust overlap between exposure and LAAsP gene signatures in both male and female mice. a. Bar graph showing the overlap between exposure and LAAsP signature DEGs in males (irrespective of direction of change). b. Matrix of Pearson’s correlation coefficients (p<0.05) between summed z-scores for exposure and LAAsP gene signature DEGs in male mice over the GTEx liver transcriptome dataset. c. UpSet plots showing overlap between LAAsP and exposure signatures for TBT, BPA.hi, and TCDD in male mice. d. Summary bar charts showing the proportion of LAAsP signature genes in the exposure signatures of males and female livers.

The observed overlap between pathways disrupted by T2C exposures shown in Figure 2 was striking given the diverse mechanisms of action of the xenoestrogen BPA, PPAR ligand TBT, and AhR agonist TCDD. This suggests that while the mechanisms of action of T2C environmental toxicants are significantly different, their ability to induce persistent changes in the transcriptome converged on a discrete set of vulnerabilities. To identify what these vulnerabilities might be, we compared the sixteen T2C exposure signatures from adult male and female livers with composite LAAsP gene signatures derived from normal 3-week and 5-month T2C male and female livers ^20^. This analysis revealed that liver LAAsP genes constituted a remarkable fraction of the T2C exposure signatures, accounting for approximately 40-60% of DEGs in exposed male and 30-60% in exposed female signatures (Figure 3a and Suppl Figure 3a). In males, the LAAsP component of the exposure signatures ranged from a low of 38% (PM2.5-JHU) to a high of 60-62% (BPA.hi and DEHP) and 43-53% of the signatures for TBT, TCDD, BPA.lo, PM2.5-Chi, and Pb.

While the enrichment for LAAsP genes in exposure signatures (30-60% of all signature DEGs) was dramatic, it was not the result of a generalized change in how the liver itself was aging. Out of 2582 LAAsP genes identified, only a fraction were reprogrammed by any one exposure. In BPA.hi for example, LAAsP genes made up 60% of the exposure signature, but these 186 DEGs were only ~7% of all 2582 LAAsP genes, and therefore not due to a generalized change in liver aging. Additionally, within exposure groups, we observed both increases and decreases in LAAsP gene expression, i.e. acceleration and attenuation of the normal gene expression trajectory, also indicating no generalized shift toward increased or decreased biological aging. Rather, the observed modulation of LAAsP genes, and their enrichment in multiple exposure signatures, suggests these genes were a susceptible target, perhaps because they retained the plasticity to change expression as a function of age, rendering them vulnerable to reprogramming by environmental exposures.

An overview of how the normal expression trajectory of LAAsP genes was disrupted by early life toxicant exposures is shown in the Pearson correlation matrices against the GTEx normal liver transcriptome, for males (Figure 3b) and females (Supplemental Figure 3b). Notably, several exposures were strongly and inversely correlated with the normal trajectory of LAAsP gene expression, indicating an attenuation of typical changes that would normally occur as the liver aged between weaning and young adulthood. For example, in the adult male liver, the BPA.hi exposure signature exhibited a strong negative correlation (r = −0.87) with normal LAAsP gene expression (i.e. attenuation of the normal expression trajectory), as did TBT(r = −0.81) (Figure 3b). In females (Supplemental Figure 3b), TBT exposure resulted in an even stronger attenuation of normal LAAsP gene expression trajectories (r = −0.99), as did PM2.5-JHU (r = −0.79), indicating an attenuated LAAsP gene signature induced by these exposures.

UpSet plots showing direction of change of TBT, BPA.hi, and TCDD signature genes compared to their normal trajectory with age are shown in Figure 3c for exposed males. LAAsP genes showing the greatest change in these exposure signatures are provided in Table 2, which illustrates how strongly early life exposures attenuated the normal age-associated increase or decrease in LAAsP gene expression. How expression of LAAsP genes changed (accelerated or attenuated) in response to all T2C exposures in both male and female mice is provided in the UpSet plots in Supplemental Figure 3c and 3d, and the complete list of LAAsP genes for all exposure signatures can be derived from the combined signatures in Supplemental Table 1.

From these analyses, an attenuated LAAsP gene signature emerged as a common feature of many T2C exposure signatures, where LAAsP genes that normally increased with age failed to do so (i.e. remained low) and those that typically decreased, remained high. Venn diagrams in Supplemental Figure 3e depict the overlap between exposure signatures and LAAsP genes in males and females, highlighting attenuation of their normal expression trajectories. The percent of LAAsP genes in each exposure signature in both males and females is summarized in Figure 3d. Thus, despite their distinct modes of action, reprogramming of the transcriptome by T2C exposures converged on a shared biological vulnerability: the inherent plasticity of genes to change expression with age.

### Histone Modifications Reveal Epigenomic Aging Patterns in the Male Mouse Liver

Histone modifications, acting as transcriptional ‘rheostats’ to finely tune gene expression, have emerged as key targets of environmental exposures ^4,21^. However, the dynamics of how histone modifications change as a function of age remain less understood compared to the plasticity of other epigenetic mechanisms, such as DNA methylation ^21–23^. Thus, we first performed ChIP-seq profiling to characterize the epigenomic programs for histone modifications over the T2C study interval, focusing on active (H3K27ac, H3K4me1, H3K4me3) and repressive (H3K27me3, H3K9me3) histone marks. These data were used to map chromatin states genome-wide, including promoters and enhancers (H3K4me3, H3K27ac, H3K4me1), as well as facultative (H3K27me3) and constitutively repressed (H3K9me3) chromatin. TaRGET II consortium-wide epigenomic profiling studies were limited to a single sex, with males selected because of their stronger phenotypic response to several T2C exposures, such as the liver tumors observed in males in response to TBT ^24,25^. During the time from weaning until 10 months of age, >350 epigenomic profiles were generated for individual exposed and vehicle control male mice.

**Figure 4:**
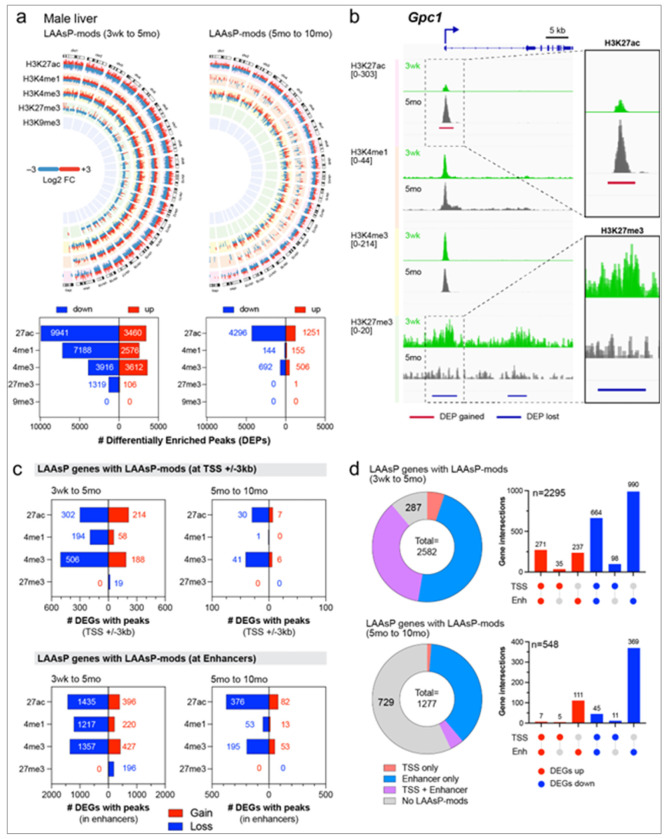
Epigenomic programming of LAAsP-mods. a. Circos plots indicating direction and magnitude of changes in histone marks with age genome-wide, and summary bar graphs. b. IGV browser LAAsP-mods at the *Gpc1* locus. c. Bar graphs showing a summary of concordance between LAAsP genes and associated LAAsP-mods at promoter regions (TSS +/− 3Kbp) and linked enhancers as determined by Enhancer Atlas. d. UpSet plots and pie charts of combinatorial LAAsP genes and associated LAAsP-mods at promoter regions and linked enhancers.

Differentially enriched peaks (DEPs) of histone modifications were identified in the liver as animals aged over two time-intervals: from weaning to early adulthood (3 weeks to 5 months) and from early to later adulthood (5 to 10 months). Analogous to LAAsP genes whose normal expression trajectory changes with age, we termed chromatin marks differentially enriched (increased or decreased) with age as liver age-associated plasticity histone modifications (LAAsP-mods). For active histone marks H3K27ac, H3K4me1, and H3K4me3, robust remodeling of the epigenome occurred during the transition from weaning to early adulthood, as illustrated by the Circos plots and bar graphs in Figure 4a, and the heatmaps for individual marks in Supplemental Figure 4a. During this period, loss of active marks predominated as weanlings aged into young adults. This reduction in active LAAsP-mods aligns with the widespread decrease in LAAsP gene expression during this time (Figure 1b). In the subsequent transition from early to later adulthood, the loss of H3K27ac and H3K4me3 marks predominated, while H3K4me1 remained relatively unchanged (Figure 4a, Supplemental Figure 4a).

The dynamics of repressive histone marks showed patterns distinct from active marks. H3K9me3, a cell-specific constitutive repressive mark, exhibited minimal change across either age interval (Figure 4a), consistent with the important role of this mark in silencing lineage-inappropriate gene expression and maintaining cell identity in the mature liver. H3K27me3, a facultative repressive mark, demonstrated broad clustering across differentially enriched regions, which contrasted to the sharp peaks characteristic of active marks (Figure 4b). H3K27me3 exhibited a sharp decline between weaning and early adulthood but was changed little between early and later adulthood (Figure 4a). This age-related decline in H3K27me3 is consistent with previous findings ^26^. However, the reported gain in that study of large H3K27me3-enriched domains in much older mice (70-95 weeks), was not observed within the 10-month timeframe of the T2C longitudinal analysis. These findings suggest that in the male liver, early loss of H3K27me3 precedes the later emergence of broad enriched domains, and the shift to what has been termed a ‘hyper-repressive state’ ^26^ occurs sometime after 10-months.

We also profiled genome-wide changes in histone marks at promoter and enhancer regions as a function of age, as detailed in Supplemental Figure 4b and Supplemental Table 3. To ensure precise linkage between genes and the enhancers known to regulate their expression, we utilized the enhancer-gene pairs cataloged by Enhancer Atlas ^27^. To do so, we first defined enhancer ‘anchors’ (Supplemental Figure 4c), where enhancers identified across different studies and tissues, but located adjacently, were consolidated into a single ‘enhancer anchor’ (Supplemental Table 4). Genes potentially regulated by each enhancer anchor were then identified using the Enhancer Atlas compendium.

While there were some gains in histone marks between weaning and early adulthood, the predominant change in LAAsP-mods was a loss of active marks in both promoter – defined as transcription start site (TSS) of genes ±3kb – and enhancer regions. Notably, however, compared to promoters, nearly an order of magnitude more LAAsP-mods occurred at enhancers (Figure 4c). Between 5 and 10 months, further loss of active marks occurred at promoters and enhancers, with enhancer regions again exhibiting significantly changes in LAAsP-mods compared to promoters (Figure 4c, Supplemental Figure 4b and Supplemental Table 3).

Enhancers are an order of magnitude more abundant than genes ^28^, implying a “many-to-one” interaction structure, where individual genes are regulated by multiple enhancers. Previous studies have shown 50% of enhancers linked to only a single gene, the average number of genes linked to a single enhancer is ~2.5, and < 1% of enhancers are highly pleiotropic (i.e. regulate multiple genes) ^29^. In contrast, while individual enhancers are linked to only a few genes, the number of enhancers that can regulate any single gene may be quite large. This is thought to help stabilize or “buffer” gene expression and provide robust enhancer regulation across multiple tissues ^29^. Our analysis of LAAsP-mods at enhancer anchors aligned well with this prediction. On average, LAAsP genes in the aging enhancer-gene network between weaning and 5 months had ~8 linked enhancers that acquired LAASP-mods (Supplemental Table 8j). Thus, between weaning and young adulthood, the observed predominance of LAASP-mods at enhancers vs promoters could be due to the fact that LAAsP genes have only a single promoter region (TSS +/− 3 kb) but orders of magnitude more enhancers capable of regulating their expression.

We next assessed the relationship between the aging epigenome and aging transcriptome by mapping LAAsP-mods for H3K4me3, H3K27ac, H3K4me1, and H3K27me3 to promoter regions and linked enhancers of LAAsP genes (H3K9me3 was not included as this mark was unchanged as a function of age) (Supplemental Table 3). Consistent with expectations, during the transition from weaning to young adulthood, LAAsP genes whose expression decreased showed loss of active histone marks (H3K27ac, H3K4me1, and H3K4me3) or gain of H3K27me3 at promoters and enhancers, while LAAsP genes whose expression increased showed gains of active marks at associated regulatory regions, or in a few cases, loss of H3K27me3 repressive mark (Figure 4c). During this interval, LAAsP-mods at enhancers were considerably more frequent compared at promoter regions of LAAsP genes (Figure 4c). In contrast, during the transition from early to later adulthood, many fewer LAAsP genes exhibited LAAsP-mods at their promoters and/or linked enhancers.

To understand age-associated combinatorial changes in epigenetic histone marks, we also generated UpSet plots for H3K27ac, H3K4me1 and H3K4me3 at promoters and linked enhancers of LAAsP genes (Supplemental Figure 4d). This analysis revealed that H3K27ac and H3K4me3 were the primary LAAsP-mods associated with epigenomic programming in the liver as male mice aged. From weaning to early adulthood, loss of H3K4me3 alone was the predominant change at promoters of LAAsP genes whose expression decreased. While many fewer LAAsP genes increased expression during this interval, these genes gained H3K4me3 and H3K27ac, alone or in combination, at their promoters. Notably, H3K4me1 showed little change at LAAsP gene promoters. At linked enhancers, coordinated loss of all three H3K27ac, H3K4me1 and H3K4me3 marks was the primary change for LAAsP genes whose expression decreased. Enhancers linked to LAAsP genes whose expression increased showed coordinate gain of all three H3K27ac, H3K4me1, and H3K4me3 marks, or H3K27ac and H3K4me3 without H3K4me1 (Supplemental Figure 4d). Between early and later adulthood, fewer LAAsP-mods mapped to promoters or linked enhancers of LAAsP genes, and during this time, gains/losses of H3K27ac, with or without changes in H3K4me3, was the predominant LAAsP-mod observed (Supplemental Figure 4b,d).

To obtain a more precise estimate of the overall association between concordant changes in the aging transcriptome and epigenome, we employed UpSet plots to classify LAAsP-mods as 1) in the promoter region (TSS ± 3 kb), 2) at a linked enhancer, 3) in both regions, or 4) in neither when marks and LAAsP gene expression were concordant (i.e decreased expression associated with the loss of active or gain of repressive marks; increased expression associated with gain of active or loss of repressive marks). As illustrated in the UpSet plots and pie charts in Figure 4d, in male livers, 2295 of the 2,582 LAAsP genes (88%) exhibited concordant changes in epigenetic histone marks in at least at one associated regulatory element between weaning and early adulthood. During the transition from early to later adulthood, when fewer LAAsP genes are differentially expressed, 42% of LAAsP genes exhibited correlative changes in LAAsP-mods. These findings reveal extensive concordance between the aging transcriptome (LAAsP genes) and programmed epigenetic aging (LAAsP-mods), especially in young adulthood.

Finally, to identify functional and cell type-specific correlates between the aging epigenome and transcriptome, we performed Over-Representation Analysis (ORA) on LAAsP genes with concordant LAAsP-mods as shown in Supplemental Figure 4e. LAAsP genes with decreased expression, and loss of active histone marks were enriched for nonparenchymal cell identity genes; LAAsP genes with increased expression and gain of active marks were enriched for hepatocyte cell identity genes (Supplemental Figure 4e). This aligns with the cell-type-specific transcriptional shifts in Figure 1e, where hepatocyte identity genes increased in expression with age, while nonparenchymal cell identity genes decreased. Additionally, ORA using the Reactome compendium revealed that LAAsP-genes that gained active LAAsP-mods enriched for metabolic pathways, whereas downregulated LAAsP genes that lost active LAAsP-mods enriched for cellular architecture and extracellular matrix production (Supplemental Figure 4e and Supplemental Table 5). These findings indicate that at both gene and pathway levels, age-related epigenomic programming of histone marks is highly concordant, with the direction, and cell-type specificity, of changes observed in the aging transcriptome of the male mouse liver.

### Early-Life Exposures Reprogram the Adult Epigenome

**Figure 5:**
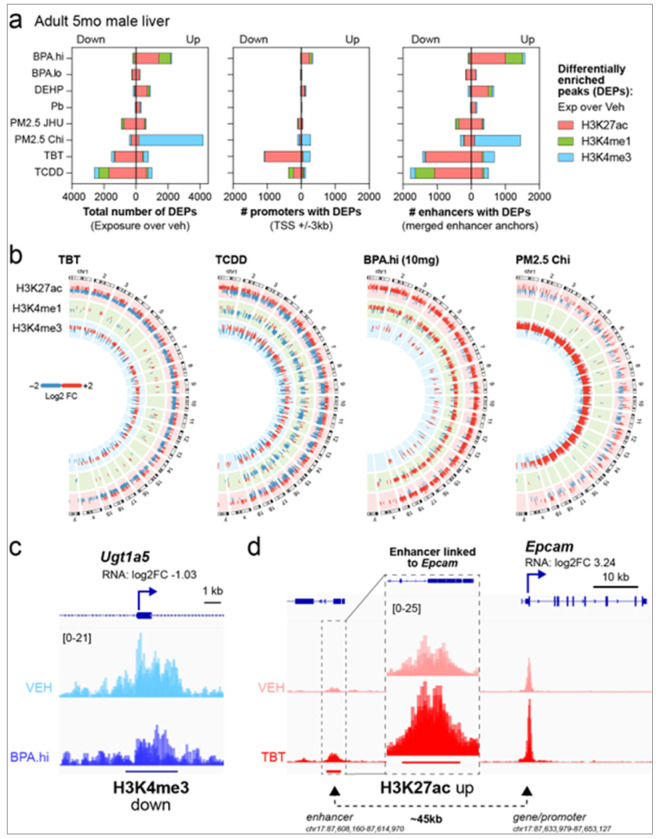
Epigenomic reprogramming induced by early-life toxicant exposures. a. Summary bar graphs for exposure-induced DEPs and their overlap with promoters and enhancers. b. Circos plots showing direction and magnitude of epigenomic reprogramming by TBT, TCDD, BPA.hi, and PM2.5-Chi. Selected IGV tracks and exposure-induced DEPs at a promoter (c) or an enhancer (d).

For each T2C exposure, exposure-induced DEPs (Figure 5a) were mapped to gene promoter (Supplemental Table 6) and enhancer (Supplemental Table 7) regions to link durable changes in epigenomic reprogramming to changes in the adult transcriptome. We found the specific histone marks affected, epigenomic states (promoters vs enhancers), and extent of the reprogramming (number of DEPs) varied dramatically among the different exposures. relative to age-matched vehicle control male mouse livers. As a result, the persistent reprogramming elicited by each T2C exposure was associated with a unique global epigenomic signature as summarized in the bar charts for all eight exposures in Figure 5a and visualized in heatmaps in Supplemental Figure 5a. For example, PM2.5-Chi predominantly induced gains in H3K4me3, with minimal changes in H3K27ac or H3K4me1. In contrast, TBT exposure caused both gains and losses of H3K27ac and H3K4me3, with losses prevailing, while leaving H3K4me1 largely unaffected. TCDD exposure caused a net loss across all three active marks – H3K27ac, H3K4me3, and H3K4me1. In the case of BPA.hi, DEPs were primarily gains in H3K27ac and H3K4me1, with minimal impact on H3K4me3. Notably, the fact that T2C exposures had distinct epigenomic signatures, as well as mechanisms of action, further reinforces that their convergent impact on the transcriptome was via targeting common vulnerabilities.

This persistent epigenomic reprogramming induced by T2C exposures was more robust at enhancers than at promoter regions for their target genes (Figure 5a). For instance, in early adulthood TCDD exposure led to loss of histone marks at >1800 enhancers vs ~300 promoter regions. The preference of T2C exposures to target enhancers over promoters for reprogramming could also be due to a high enhancer:promoter ratio per target gene, although the possibility exposures preferentially target enhancers compared to promoter regions (i.e, enhancers are more susceptible to reprogramming) or that when reprogrammed, DEPs in enhancers are more enduring than those in promoter regions (i.e. enhancer reprogramming is more persistent) should not be ruled out.

These findings underscore that early-life T2C exposures drive durable, exposure-specific epigenomic reprogramming in the adult male liver, which varies markedly in both the number, direction, and specific epigenetic marks affected. This variability in epigenomic reprogramming is further illustrated with Circos plots for TCDD, TBT, BPA.hi, and PM2.5-Chi (Figure 5b), and other exposures in Supplemental Figure 5b. However, while some early-life T2C exposures induced robust changes in the adult liver epigenome, others had minimal effect. T2C exposures ranked for extent of reprogramming (as determined by number of DEPs), were PM2.5-Chi > TCDD, TBT, and BPA.hi > PM2.5-JHU and DEHP > Pb and BPA.lo (Supplemental Tables 6 and 7).

The persistent epigenomic reprogramming induced by T2C exposures was not merely a consequence of altered gene expression, as extensive reprogramming also occurred in promoter regions and linked enhancers of genes where expression was unchanged in the exposed livers, a phenomenon previously referred to as “silent reprogramming” ^30^. This is evident from the disproportionate number of reprogrammed promoters and enhancers relative to exposure-induced DEGs for several exposures. For example, TBT exposure resulted in the loss of H3K27ac at over 1,000 promoters and more than 1,200 enhancers, yet its adult liver exposure signature had only 354 DEGs (compare Figure 5a and Figure 2a). Similarly, BPA.hi increased H3K27ac and H3K4me1 at more than 200 promoters and over 1,500 enhancers but had only ~300 signature DEGs. PM2.5-Chi exposure induced gains in H3K4me3 at over 200 promoters and more than 1,500 enhancers (>1,700 DEPs) but with an exposure signature of just 478 DEGs. This “silent reprogramming” mirrors findings from perinatal BPA exposure in rats ^30^, where early-life BPA left a persistent epigenetic fingerprint on target genes that “primed” them for aberrant expression only in response to later-life stressors. In that study, epigenetic reprogramming of EGR1 target genes by BPA led to exaggerated transcriptional activation under high-fat diet conditions, ultimately causing metabolic dysfunction. These results suggest that even the silent epigenomic changes observed in response to T2C exposures could serve as a latent mechanism for maladaptive responses to other stressors later in life.

To link signature DEGs to exposure-induced epigenomic reprogramming, we mapped exposure-induced DEPs to the promoter and enhancers regions for each DEG in the male exposure signature (Supplemental Table 8). UpSet plots summarizing the overlap between DEGs and DEPs in promoter or enhancer regions are shown in Supplemental Figure 5c and d. Between 26-37% of promoters and/or linked enhancers of signature DEGs exhibited persistent epigenomic reprogramming in livers of mice exposed to BPA.hi, TBT, TCDD, and PM2.5-Chi (Table 3). This association became even more pronounced when the direction of the global epigenomic response to each exposure (Figure 5a) was aligned with the direction of DEG expression. For instance, BPA.hi primarily induced genome-wide gain in active marks such as H3K27ac, H3K4me3, and H3K4me1, and 53% of BPA.hi DEGs whose expression was increased were linked to gains in these marks at their promoters and/or enhancers. Similarly, TBT predominantly drove loss of H3K27ac and H3K4me1, and 51% of DEGs exhibiting decreased expression were associated with losses in these marks. In contrast, exposures with minimal impact on the epigenome, PM2.5-JHU, BPA.lo, DEHP, and Pb, exhibited the fewest signature DEGs and only limited epigenomic reprogramming of promoter or linked enhancer regions (5–16%; Table 3).

This relationship extended to LAAsP genes in the exposure signatures of BPA.hi, TBT, TCDD, and PM2.5-Chi. Examples of this reprogramming are illustrated in the IGV tracks for *Ugt1a5*, which lost H3K4me3 at the promoter and was decreased in the BPA.hi signature (Figure 5c), and *Epcam*, which gained H3K27ac at its linked enhancer, and was increased in the TBT signature (Figure 5d). Additional examples are provided in Supplemental Figure 5e-f for genes reprogrammed and differentially expressed in response to PM2.5.Chi, TCDD, and TBT. As shown in Table 3, 25–40% of LAAsP genes in these exposure signatures displayed epigenomic reprogramming at promoters and/or linked enhancers, further underscoring the interplay between exposure-induced epigenetic alterations and persistent reprogramming of the adult transcriptome (Supplemental Table 8). Taken together, the picture that emerged for BPA.hi, TBT, TCDD, and, to a lesser extent, PM2.5-Chi, was that despite differences in their modes of action, these early-life exposures induced significant epigenomic reprogramming at enhancers linked to their signature DEGs. Thus, enhancers are a key target for the long-lasting effects of early-life exposures, playing a much larger role in epigenomic reprogramming than previously appreciated.

### Early-Life Exposures Polarize the Adult Transcriptome with Cell-type and Directional Specificity

In the liver, metabolically active hepatocytes constitute approximately two-thirds of cells, while non-parenchymal cells, such as Kupffer, Stellate, and sinusoidal endothelial cells, make up the remaining third, each contributing to liver structure and function in unique ways ^31^. To explore how exposure-induced reprogramming impacts specific liver cell types, we conducted GSEA on the transcriptomes of both male and female mice using identity genes for epithelial parenchymal cells (hepatocytes and bile duct epithelial cells/cholangiocytes) and various non-parenchymal cell types, including Stellate, Kupffer, endothelial, and resident immune cells (B-cells and NK-NKT cells). This analysis revealed a remarkable polarization of the transcriptome induced by TBT, TCDD, BPA.hi, and PM2.5-Chi, with pathway enrichment for liver cell identity genes for all four exposures exhibiting striking directionality and an attenuated LAAsP gene signature.

**Figure 6:**
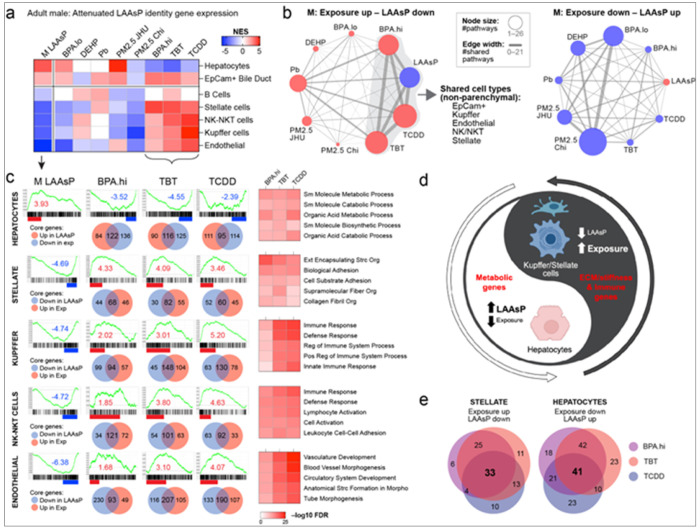
Early life exposures polarize the liver transcriptome. a. GSEA enrichment results showing attenuation of normal gene expression trajectories in young adult males using 7 cell-identity signatures from the Aizarani liver compendium. b. Network analysis of non-parenchymal cell identity vs LAAsP signature genes. c. GSEA enrichment plots with Venn diagrams for overlaps between LAAsP and exposure core genes from 5-month males exposed to BPA.hi, TBT, and TCDD in Stellate, Kupffer, MVECs, LSECs, and Hepatocytes. GSEA core genes were further analyzed using ORA for enriched gene sets from the GOBP compendia. d. Inverse relationship between aging processes and cell-type specificity derived from T2C exposure signatures. e. Venn diagrams for core gene overlap in livers exposed to TBT, TCDD, and BPA.hi for hepatocyte and Stellate cell-identity genes.

Identity genes for the 32 liver-resident cell types ^32^ (Supplemental Figure 6a) were grouped into two parenchymal (hepatocytes and bile duct epithelial) and five non-parenchymal (Stellate, Kupffer, NK, vascular, and B cells) categories, based on their collective identity genes (Figure 6a, males and Supplemental Figure 6b, females). Although we anticipated cell-type specificity in gene expression changes caused by T2C exposures, an unexpected finding was the clear directionality, and pronounced anti-correlation, with normal LAAsP gene expression trajectories in all cell types of both male and female livers. In males exposed to TCDD, TBT, BPA.hi, and PM2.5-Chi, expression of hepatocyte LAAsP genes that normally increased from weaning to young adulthood was attenuated, remaining low relative to age-matched 5-month-old controls. In PM2.5-Chi exposed mice, a similar attenuation was observed in bile duct epithelial cells (Figure 6a). Remarkably, non-parenchymal cells also showed attenuation of normal LAAsP gene expression trajectories, but opposite that seen for parenchymal cells. In nonparenchymal cells, in response to TCDD, TBT, and BPA.hi, LAAsP genes that typically decreased from weaning to young adulthood remained high in sinusoidal endothelial, Kupffer, Stellate, and resident immune cells in the male liver relative to age-matched 5-month-old controls (Figure 6a).

In females, a similar attenuated aging effect, characterized by suppressed expression of hepatocyte LAAsP genes was observed in response to PM2.5-JHU, BPA.lo, Pb, TBT, and TCDD. In bile duct epithelial cells, this effect was also evident in response to BPA.hi, PM2.5-JHU, and TCDD. Conversely, as seen in males, non-parenchymal cell LAAsP genes that usually declined with age remained high in livers exposed to BPA.lo, Pb, TBT, TCDD, and PM2.5-Chi (Supplemental Figure 6b).

Network diagrams show the extent to which LAAsP gene expression trajectories were attenuated in various non-parenchymal liver cells. These diagrams depict the extent of overlap between nonparenchymal cell identity LAAsP genes that normally decreased (blue node) or increased (red node) and those in the T2C exposure signatures. Edge thickness denotes the degree of overlap between LAAsP and exposure signature genes. As shown for males (Figure 6b) and females (Supplemental Figure 6c), there was extensive overlap between LAAsP genes (blue node) and signature DEGs (red nodes) —albeit in the opposite direction— for multiple non-parenchymal cell types. Notably, however, as described above, this was the case only for those LAAsP genes that normally decreased between weaning and adulthood. For example, in males, LAAsP genes that typically decrease with age (shown as the blue node in “Exposure up” network diagram in Figure 6b) remain elevated (red nodes) in many non-parenchymal cell types following exposure to BPA.hi, TCDD, and TBT. There were fewer non-parenchymal cell identity genes that increased with age (note the small red aging node in the “Exposure down” network diagram in Figure 6b), and these genes showed little overlap with any T2C exposure signatures, despite the fact that several signatures contained large numbers of identity genes that were decreased (note the large blue exposure nodes in that diagram). A similar pattern emerged in females, where the most pronounced reprogramming of non-parenchymal cell types occurred in response to TCDD, PM2.5-Chi, and TBT, resulting in elevated expression of LAAsP genes that would have normally decreased with age (Supplemental Figure 6c). Thus, in both sexes, reprogramming induced by multiple exposures exhibited dramatic cell-type specificity and directionality, resulting in a polarized attenuated aging signature of increased expression of LAAsP genes in nonparenchymal cells and decreased expression of LAAsP genes in parenchymal cells.

For T2C exposures that had the greatest impact on the adult transcriptome, we also conducted GSEA to ask if there were functional correlates for the LAAsP signature genes whose expression trajectory had been attenuated in parenchymal and non-parenchymal cells. In males, Figure 6c shows enrichment plots for cell-identity genes that change with age (far left panels) in hepatocytes, stellate, Kupffer, NK-NKT, and endothelial cells, and enrichment plots for the exposure signatures of BPA.hi, TBT, and TCDD (right). Taking the core genes from each enrichment plot, we utilized Venn diagrams to find the overlap between cell identity LAAsP genes that normally changed with aging and DEGs in the exposure signatures. In males exposed to BPA.hi, TBT, and TCDD, for cell identity genes of both parenchymal and nonparenchymal cells, we consistently observed an anti-correlation between core genes in the LAAsP and exposure signatures, i.e attenuated aging trajectories. Specifically, for hepatocytes, core LAAsP cell-identity genes that typically increase with age showed significant overlap with exposure core DEGs that decreased—reflecting attenuated aging—in adult males exposed to BPA.hi, TBT, and TCDD (Figure 6c). Conversely, in non-parenchymal cells, we found substantial overlap between core LAAsP identity genes that decreased in Stellate, Kupffer, NK, and endothelial cells with signature DEGs that were significantly increased in response to TBT, TCDD, and BPA exposures (Figure 6c). This attenuated aging effect was similarly observed in females (Supplemental Figure 6d).

In males for example, 206 core hepatocyte LAAsP genes typically increased between weaning and young adulthood (normalized enrichment score [NES] = 3.93), and among these, expression was attenuated for 122 (59%; NES = −3.52), 116 (56%; NES = −4.55), and 95 (46%; NES = −2.39) in adult hepatocytes exposed to BPA, TBT, and TCDD, respectively. A similar attenuated aging was observed in non-parenchymal cells. In Stellate cells, 112 core genes normally decreased with age (NES = −4.89), of which 68 (61%; NES = 4.33), 82 (73%; NES = 4.09), and 60 (54%; NES = 3.46) remained high in livers exposed to BPA, TBT, and TCDD, respectively. Similar overlaps were seen for core genes in Kupffer, NK-NKT, and endothelial cells, where LAAsP cell identity genes that typically decreased with age remained elevated in adult livers of exposed males (Figure 6c) and females (Supplemental Figure 6d). Sex-specific responses were also observed. For instance, PM2.5-Chi attenuated expression of LAAsP hepatocyte cell identity genes only in males, and TCDD attenuated expression of LAAsP cell identity genes in Stellate and endothelial cells only in male livers (compare Figures 6a and Supplemental Figure 6b).

We next utilized the genes in the intersection of aging and exposure signatures to perform ORA to identify functional pathways enriched in the attenuated aging genes for different liver cell types in males exposed to BPA.hi, TBT and TCDD (Figure 6d) and females exposed to PM2.5-Chi, TBT and TCDD (Supplemental Figure 6d, Supplemental Table 9b-c). Note, GSEA looks at the entire transcriptome, and Core genes are defined as those that contribute the most to the enrichment signal and therefore may not all meet the same criteria for DEGs (i.e. changed by at least 1.5-fold with FDR<0.05). Therefore, numbers of Core genes will generally be greater than number of DEGs, which are used for ORA. In hepatocytes, LAAsP cell identity genes predominantly enriched for metabolic pathways that typically increase with age, but which remained low in the adult liver of exposed animals. Conversely, in non-parenchymal cells, attenuated LAAsP cell identity genes were enriched for pathways associated with cytoskeletal organization, extracellular matrix production, and immune signaling, which rather than decreasing with age, remained high in exposed animals. This inverse relationship, wherein the normal expression trajectory for LAAsP gene expression becomes attenuated in response to T2C exposure, with cell-type and directional specificity, is depicted schematically in Figure 6d, highlighting the dynamic interplay between epigenomic reprogramming and cell-type-specific aging trajectories, and the systemic impact of these early-life exposures across multiple cell types.

To confirm that the observed attenuation of cell identity gene expression was due to disruption of the transcriptome rather than shifts in liver cell composition, we employed MuSiC (Multi-subject Single-cell deconvolution) analysis (Supplemental Figure 6e) to assess the impact of T2C exposures on composition of liver parenchymal and non-parenchymal cells. None of the exposures induced significant changes in the proportions of these cell types, with the exception of PM2.5-Chi, where a slight, but statistically significant increase in the proportion of hepatocytes occurred. Notably, this minor change in cell composition was opposite to the transcriptional reprogramming observed where hepatocytes (despite their increase in number) showed a reduction in metabolic gene expression. This confirms that the phenotype of attenuated of LAAsP gene expression was driven by exposure-related reprogramming rather than any change in cellular proportions.

We also asked if exposures reprogrammed the same, or different, cell identity genes for specific liver cell types. As shown using heatmaps in Supplemental Figure 6f for core identity genes and Venn diagrams in Figure 6e, we identified LAAsP genes reprogrammed by one or more exposures in hepatocytes or Stellate cells (see Supplemental Table 9). The picture that emerged for BPA.hi, TCDD, and TBT in hepatocytes was that only about a quarter (41/178) of the hepatocyte LAAsP cell identity genes reprogrammed in the adult male liver were targeted by all three exposures. Similarly in Stellate cells, about a third (33/102) of LAAsP Stellae cell identity genes were targeted by all three exposures. This reinforces that pathways with age-related plasticity **—such as hepatocyte metabolism and stellate cell extracellular matrix production—are prime targets for reprogramming by early-life exposures, regardless of toxicant mechanism, epigenetic signature, and in many cases specific pathway genes affected.**

### A LAAsP Gene Signature Distinguishes Healthy from Diseased Liver in Patient Populations

**Figure 7:**
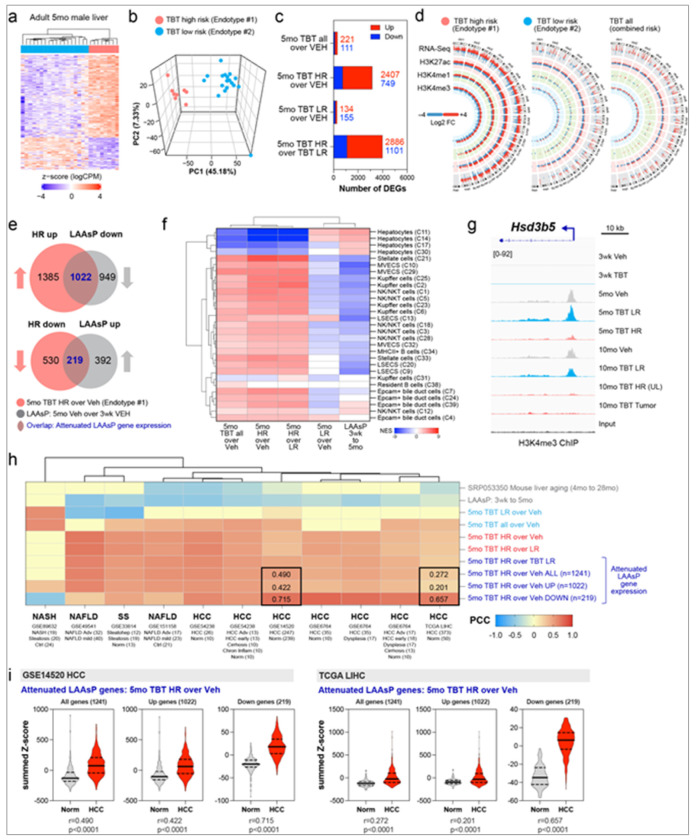
LAAsP genes attenuation and exposure signatures distinguish healthy from diseased livers. a. Heatmap and b. PCA of Endotype 1 (high risk/HR) and Endotype 2 (low risk/LR) TBT exposure signatures in young adult male mice. c. Summary of exposure DEGs in HR and LR. d. Circos plots showing direction and magnitude of exposure DEPs in the HR and LR endotypes compared to age matched vehicle controls, contrasted to all TBT-exposed animals combined e. Attenuation of LAAsP gene expression in the TBT HR signature. f. GSEA enrichment of liver cell-identity genes in liver signatures for young adult TBT-exposed mice and LAAsP gene-expression signatures. g. IGV plot showing Endotype-specific LAAsP-mods at the *Hsd3b5* locus. h. Pearson Correlation Coefficient analysis of TBT-exposure and LAAsP gene signatures against multiple human liver disease cohort transcriptomes (p<0.05). i. Summed z-score distribution using the up-regulated, down-regulated, or combined gene signature from the TBT HR endotype for two human liver cancer transcriptomic datasets (p<0.05).

Non-alcoholic fatty liver disease (NAFLD) has been linked to environmental exposures, and is a precursor to hepatocellular carcinoma (HCC) ^33–35^. In the T2C studies, TBT exposure induced NAFLD in male mice ^36,37^ and, with variable penetrance, liver tumors at 10 months ^24,25^. Uninvolved liver tissue from tumor-bearing mice had a distinct “high-risk” epigenomic/transcriptomic signature similar to tumors, whereas liver tissue from non–tumor-bearing mice had a “low-risk” signature similar to vehicle controls, despite being identically exposed to TBT. Variable tumor penetrance was thus linked to heterogeneity in EDC-induced liver reprogramming. Even as early as 5 months, prior to development of tumors, livers from young adult TBT-exposed males segregated into two endotypes: Endotype 1, mirroring the high-risk signature, and Endotype 2, resembling the low-risk signature (Fig. 7a–b) ^25^. Blood transcriptomes confirmed TBT exposure in both endotypes, consistent with the systemic maternal exposure paradigm ^25^.

Comparative analyses of the liver transcriptomes and epigenomes of young adult Endotype 1 and Endotype 2 mice, relative to age-matched vehicle controls, revealed striking differences (Figure 7c–d and Supplemental Table 10). Endotype 1 (similar to the high-risk livers) exhibited a markedly distinct transcriptional profile, with 2,407 upregulated and 749 downregulated DEGs relative to vehicle controls. In contrast, Endotype 2 (similar to the low-risk livers) more closely resembled vehicle controls, with only 134 upregulated and 155 downregulated DEGs. This pronounced divergence highlights the robust signature of Endotype 1 transcriptomes, compared to data aggregated for 5-month-old animals where 221 were significantly up- and 111 were significantly down-regulated (Figure 7c), and underscores the impact of molecular heterogeneity on exposure signatures. It also demonstrates the critical value of profiling individual animals, which when analyzed individually rather than with pooled data, can revealed distinct signature endotypes, in this case, present in exposed animals prior to tumor development.

The epigenomic profiles of Endotype 1 and Endotype 2 your adult livers were similarly distinct, as shown in the Circos plots in Figure 7d. Endotype 1 livers exhibited significant epigenomic divergence from age-matched vehicle controls, whereas Endotype 2 livers showed minimal changes. As shown in Figure 7d, Endotype 1 livers exhibited substantially greater gains and losses in H3K27ac, H3K4me1, and H3K4me3 compared to Endotype 2 livers, further highlighting the critical differences between these two response endotypes. Importantly, when epigenomic profiles from all 5-month TBT-exposed mice were aggregated, the overall response appeared muted, masking the pronounced changes that had occurred in Endotype 1 animals (Figure 7d).

As described above, in parenchymal cells LAAsP genes normally increase with age, while in nonparencymal cells, LAAsP genes decrease. Appreciating that for attenuated LAAsP gene expression, the direction of change induced by exposures reflects reprogramming of specific cell types, we also looked at attenuated expression of LAAsP genes in the Endotype 1 (high-risk) livers. 42% of Endotype 1 signature DEGs that were upregulated (1022/2407), and 29% of signature DEGs that were downregulated (219/749), reflecting reprogramming of nonparenchymal and parenchymal cells, respectively, overlapped with LAAsP signature genes (Figure 7e). GSEA for parenchymal and nonparenchymal cell identity genes, as shown in the NES heat map in Figure 7f, revealed the polarized attenuated LAAsP gene expression phenotype seen with aggregated data was clearly driven by reprogramming in the Endotype 1 livers, as detailed in Supplemental Table 11. In the heatmap in Figure 7f, across parenchymal and nonparenchymal cell types, Endotype 1 with “high-risk” transcriptomic and epigenomic features, whether compared to vehicle or Endotype 2 “low-risk” livers, formed a distinct cluster. The high-risk TBT Endotype 1 signature was robustly anti-correlated with expression trajectories of both parenchymal and nonparenchymal cell-identity genes (Figure 7f). In contrast, the TBT Endotype 2 low-risk signature clustered separately and was aligned directionally with the normal expression trajectories of both parenchymal and nonparenchymal cells (Figure 7f).

An example of the epigenomic correlates for this reprogramming is shown in Figure 7g for the hepatocyte metabolic gene hydroxy-delta-5-steroid dehydrogenase, 3 beta- and steroid delta-isomerase 5 (*Hsd3b5*). Between weaning to early (5 months) and later (10 months) adulthood, *Hsd3b5* expression in hepatocytes normally increases 16- to 64-fold, accompanied by gain of H3K4me3 at the *Hsd3b5* in the promoter region, identifying this as LAAsP-mod linked to *Hsd3b5* expression (Figure 7g). The gain of H3K4me3 seen in vehicle controls with age also occurs in Endotype 2 TBT-exposed livers at 5 months, and in low-risk 10-month TBT-exposed livers of animals that did not develop tumors. However, H3K4me3 was absent in livers of 5-month-old Endotype 1 and uninvolved liver tissue of 10-month-old high-risk mice that developed liver tumors. In these tissues, *Hsd3b5* expression was ~100-fold less than what is seen in age-matched controls. Thus, heterogeneity in response is also reflected in reprogramming of LAAsP-mods (i.e. H3K4me3) in response to early life exposures linked to attenuation of age-appropriate epigenomic programming and expression of LAAsP genes (i.e. *Hsd3b5*).

To establish the translational relevance of attenuation of LAAsP gene expression signatures, we next performed Pearson’s correlation using transcriptomic data from multiple clinical liver diseases datasets: NAFLD, Non-Alcoholic Steatohepatitis (NASH), simple steatosis (SS), and HCC. These patient-derived liver transcriptomes were compared to the normal mouse LAAsP gene signature between weaning and young adulthood, and a publicly available liver aging gene set for mice between 4 and 28 months (PMC4636074), the TBT Endotype 1 (high-risk) and Endotype 2 (low-risk) exposure signatures. Strikingly, the transcriptomes of human liver disease patients were strongly and negatively correlated with normal liver aging (Figure 7h), similar to what was observed in T2C study animals for exposures that attenuated the expression trajectory of LAAsP signature genes. Equally remarkable was the strong positive correlation between the TBT Endotype 1 (high-risk) signature and the transcriptomes of liver disease and HCC patients. In the case of HCC, this correlation was strongest for the LAAsP gene component of the high-risk TBT Endotype 1 signature: genes that were downregulated and enriched for hepatocyte identity genes (Figure 7h).

Further analysis of the downregulated component of the Endotype 1 signature using GSEA for hepatocyte cell-identity genes (recall again that GSEA Core genes can often exceed the number of DEGs) identified 302 negatively enriched core genes across all four classes hepatocytes (Suppl Figure 7a and 7b). Transcription factor motif analysis showed these core genes were significantly enriched for several transcription factors, with the most enrichment seen for NHR1H4, the bile acid farnesoid X receptor (FXR) (NES= log10-^17^) (Suppl Figure 7c). FXR is well known to play a key role in age-associated changes in the liver transcriptome, as well development of liver disease and HCC ^38,39^, suggesting a combined effect of epigenomic reprogramming (Figure 7d) and altered transcription factor activity (Suppl Figure 7c) as potential drivers for the increased risk for liver tumors associated with this high risk endotype.

Focusing on the transcriptomes for tumor and non-tumor tissue from HCC patients (GEO GSE14520 and TCGA), LAAsP signature genes from the high-risk Endotype 1 TBT exposure group that were enriched for hepatocyte cell identity genes (i.e. decreased-see Figure 7f) most effectively distinguished the transcriptomes of normal vs diseased livers (Figure 7i). In the GEO GSE14520 dataset, LAAsP signature genes that *decreased* in the Endotype 1 high-risk signature showed the most robust correlation with HCC (r = 0.715), compared to LAAsP signature genes that *increased* (i.e. enriched for nonparenchymal cell-identity genes) (r = 0.422) or when all genes were aggregated (r = 0.490) (Figure 7i left). Similar analysis of TCGA dataset confirmed this association: LAAsP signature genes that *decreased* in the high-risk TBT signature better differentiated normal liver from HCC (r = 0.657) than genes that were increased (r = 0.201) or all genes aggregated (r = 0.272) (Figure 7i right). These findings point to a fundamental link between attenuation of LAAsP gene expression trajectories and liver disease, including HCC, a linkage conserved across species, and vulnerable to disruption by early-life environmental exposures.

In summary, the NIEHS TaRGET II Consortium undertook the most comprehensive parallel transcriptomic and epigenomic profiling effort to date to shed light on the persistent effects of early-life environmental exposures. These studies revealed that in the liver, epigenetic histone modifications – particularly those associated with enhancers – and age-associated gene expression trajectories are key targets for reprogramming by diverse early-life environmental insults. The findings suggest that the inherent plasticity required for programmed epigenomic aging, along with shifts in the trajectory of aging-associated gene expression, underpins the liver’s vulnerability to reprogramming by environmental exposures. Crucially, aberrant expression of LAAsP genes, particularly attenuation of their normal aging trajectory, was linked to risk for development of liver tumors in mice and correlated with development of liver disease and HCC in patients. Together, these findings provide groundbreaking insights into the long-lasting effects of early life exposures on the epigenome and transcriptome and demonstrate how environmental exposures can be used as a powerful tool to obtain new insights into fundamental mechanisms driving human disease.

## MATERIALS AND METHODS

### Animal exposure paradigm and tissue collection

C57BL/6 (B6; Jackson Laboratory, Bar Harbor, ME) mice were used for all experiments, except for the lead (Pb) and phthalate (DEHP) exposure studies, which employed wild-type non-agouti (a/a) mice derived from a >230-generation colony of viable yellow agouti (Avy) mice. These a/a mice are genetically invariant and exhibit 93% genetic identity with the C57BL/6J strain ^40^. Mice were exposed to toxicants perinatally via maternal diet, drinking water, or air breathing, with the exposure window spanning from pre-conception through weaning. A schematic overview of the experimental design is provided in Supplemental Figure 1a. In brief, two weeks before mating, virgin female dams (6–8 weeks old) were randomly assigned to one of nine exposure groups or a control group:

1. 50 mg/kg diet of BPA (BPA 10mg);
2. 50 μg/kg diet of BPA (BPA 10ug);
3. 25 mg/kg diet of DEHP (DEHP);
4. 32 parts per million (ppm) Pb-acetate drinking water (Pb);
5. PM <2.5 micrometer (PM2.5-JHU);
6. PM <2.5 micrometer (PM2.5-CHI);
7. 0.5 mg/kg BW tributyltin (TBT) drinking water;
8. 1 ug/kg per BW of dioxin (TCDD) via oral gavage;
9. 10 parts per billion (ppb) sodium arsenite drinking water (As).

Female mice (dams) were mated with virgin males (7–9 weeks old) following two weeks of exposure to the assigned experimental diets or water. Exposure continued throughout pregnancy and lactation until offspring were weaned at postnatal day 21 (PND21). Animals were housed in polycarbonate-free cages under a 12-hour light/dark cycle. All experimental procedures were approved by the Institutional Animal Care and Use Committees (IACUC) at each participating production site and were conducted in accordance with the guidelines established by the NIEHS TaRGET II Consortium, adhering to the highest standards of animal welfare ^2^. Numbers of mice in each arm used for the liver analysis in this study are shown in Table 4 below.

As, Pb, and TBT exposures were administered via drinking water provided *ad libitum*. BPA (high and low dose) and DEHP were administered via diet provided ad libitum. TCDD was administered via oral gavage. PM2.5 exposures were administered via a breathing control chamber as scheduled.

### BPA exposure

For BPA exposure, a modified AIN-93G diet supplemented with 50 mg/kg BPA (TD.06156; Harlan Teklad) was used for the high-dose group, and a modified AIN-93G diet supplemented with 50 μg/kg BPA (TD.110337; Harlan Teklad) was used for the low-dose group, as previously described ^41^. All diet ingredients were sourced from Harlan Laboratories (Madison, WI), with BPA obtained from Sigma-Aldrich (St. Louis, MO). Drinking water was provided in polypropylene bottles. The estimated daily BPA intake was approximately 10 mg/kg body weight (bw) for the high-dose group and 10 μg/kg bw for the low-dose group. Throughout the experiment, animals were maintained on a phytoestrogen-free modified AIN-93G chow (Envigo TD.95092; 7% corn oil diet, Harlan Teklad).

### DEHP exposure

DEHP was dissolved in corn oil (Envigo) to prepare a stock solution used to generate a customized 7% corn oil chow for experimental exposures. The DEHP concentration was selected to achieve a target maternal dose of 5 mg/kg/day, based on an estimated body weight of 25 g and a daily chow intake of 5 g for pregnant and nursing female mice. This dosing strategy was informed by previous studies demonstrating obesity-related phenotypes following perinatal DEHP exposure at similar levels ^42^, and falls within the range of human exposure levels ^43^. Throughout the experiment, animals were maintained on phytoestrogen-free modified AIN-93G chow (Envigo Td.95092, 7% corn oil diet, Harlan Teklad).

### Pb exposure

Pb-acetate drinking water was prepared by dissolving Pb(II) acetate trihydrate (Sigma-Aldrich) in distilled water to a final concentration of 32 ppm, modeling human-relevant perinatal exposure levels. Previous studies confirmed that this dosing regimen results in maternal blood lead levels (BLLs) ranging from 16 to 60 μg/dL (mean: 32.1 μg/dL) ^44^. All exposure water was prepared in a single batch, and Pb concentrations were validated using inductively coupled plasma mass spectrometry (ICP-MS; NSF International; limit of detection = 1.0 μg/L). Animals were maintained on phytoestrogen-free modified AIN-93G chow (Envigo Td.95092, 7% corn oil diet, Harlan Teklad) for the duration of the experiment.

### TBT exposure

For TBT exposure, animals were administered tributyltin (TBT) chloride (96% purity; Sigma-Aldrich) via drinking water, as previously described ^24^. Water consumption was surveyed in non-pregnant females to estimate the administered TBT dose. Consumption was found to increase sharply for approximately three days following parturition, during the onset of lactation. Based on an average intake of 10 mL/day, animals received 200 mL of 3.07 μM TBT solution weekly, corresponding to an estimated dose of 0.5 mg/kg body weight/day. This dose falls below the lowest range of the developmental no observed adverse effect level (NOAEL) (5.8–20 mg/kg body weight) as defined by the Concise International Chemical Assessment Documents (CICADs) for developmental toxicity ^45^. Animals were maintained on phytoestrogen-free modified AIN-93G chow (Envigo Td.95092, 7% corn oil diet, Harlan Teklad) throughout the study.

### TCDD exposure

For TCDD exposure, female mice were administered 2,3,7,8-tetrachlorodibenzo-p-dioxin (TCDD; Cambridge Isotopes, cat# ED-901-C) dissolved in corn oil via oral gavage. Dams received three doses of 0.34 μg/kg body weight each, totaling 1 μg/kg body weight across the exposure period from preconception through weaning. Dosing occurred at three time points: two weeks prior to mating, approximately embryonic day 7, and approximately postnatal day 14. Control animals received pure corn oil. Throughout the study, all animals were maintained on phytoestrogen-free modified AIN-93G chow (Envigo Td.95092, 7% corn oil diet, Harlan Teklad).

### PM2.5-JHU exposure

Animals were exposed to either PM2.5 or filtered air (FA) in dedicated exposure chambers. Exposure occurred 5 days per week (Monday through Friday) from 9:00 AM to 5:00 PM, for a total of 8 hours per day, over a period of 7 weeks. Prior to exposure, all animals were acclimated in the housing facility for one week. Throughout the 7-week exposure period, each group of mice (FA or PM2.5) was rotated between two separate exposure chambers to minimize potential chamber-specific effects. No significant differences in locomotor activity or mobility were observed in any of the mice prior to or during the exposure period, ensuring that baseline physical activity was consistent across groups. Mice were provided with control water and food ad libitum throughout the duration of the PM2.5 exposure.

### PM2.5-CHI exposure

Dams were placed in either the PM or control chamber for 8 hours daily and mated overnight with male mice until pregnancy was confirmed, as previously described ^46^. Upon confirmation of pregnancy, dams continued exposure to either PM2.5 or filtered air for 8 hours per day until the pups were born. Both dams and pups were then exposed to their respective conditions (PM2.5 or filtered air) for 8 hours per day until the pups reached 3 weeks of age.PM2.5 was concentrated from ambient air in Chicago using a chamber connected to the Versatile Aerosol Concentration Enrichment System (VACES), as described previously ^47^. The chemical composition of the PM2.5 used in this study has been previously reported ^47^. Control mice were exposed to filtered air in an identical chamber connected to the VACES, where a Teflon filter was placed on the inlet valve to remove all particles. Mice were provided with control water and food ad libitum throughout the duration of the PM2.5 exposure.

At postnatal day 21 (PND21), all offspring were weaned and transitioned to control water and control chow. Consortium-wide, one set of male and female mice from each exposure group was euthanized at 3 weeks of age for tissue collection to perform molecular analyses. A second set of males and females were maintained until 5 months of age, and a third set of males and females were maintained until 10 months of age. This study evaluates animals collected at all three time points (3 weeks, 5 months, and 10 months of age).

Blood, liver, cortex, heart, lung, and other tissues were collected from all animals. All animals and collected tissues were included in subsequent analyses, with no exclusions necessary. For organ and tissue collection, mice were fasted for four hours during the light cycle, beginning in the morning. In the afternoon, animals underwent CO2 euthanasia followed by cardiac puncture, and whole-body perfusion with saline (Sigma). The cortex and liver were then dissected, immediately flash frozen in liquid nitrogen, and stored at −80°C. Tissue collection and processing followed protocols established by the TaRGET II Methods Working Group. Blood was collected in EDTA-coated tubes (Fisher, Cat. #365974), and plasma was separated by centrifugation. Red blood cells were lysed using erythrocyte lysis solution (Qiagen, Cat. #79217), and the remaining white blood cells were washed in PBS and resuspended in Buffer RLT (Qiagen, Cat. #79216) for subsequent DNA extraction. Standardized operating procedures (SOPs) for the consortium’s tissue collection can be accessed on our website at https://targetepigenomics.org/documents/, including SOP_Blood_Processing, SOP_Mouse_Liver_Collection, and SOP_Whole_Mouse_Perfusion. For each mouse, one investigator administered the treatment and was therefore aware of the treatment group allocation. All investigators performing subsequent molecular assays were blinded to the treatment group until the treatment groups were revealed during bioinformatics analyses.

### RNA extraction and RNA-seq library construction

Total RNA was isolated via TRIzol Reagent (Thermo Fisher Scientific, 15596026), Phasemaker Tubes (Thermo Fisher Scientific, A33248) and RNA Clean & Concentrator-5 (Zymo Research, R1013). In brief, rat brain tissues were homogenized in 1 ml of TRIzol Reagent, the tissue lysates were transferred to a pre-spined Phasemaker Tube. 0.2 ml of chloroform was added to the tube, the tubes were then shaken for 15 s. The mixture was centrifuged at 16,000 g for 5 min, the top aqueous phase was transferred to a microcentrifuge tube. An equal volume of ethanol was added to the aqueous phase. The RNA purification with DNase treatment was performed following the manual of the RNA Clean & Concentrator kit, with Ribosomal RNAs were removed from 500 ng for the total RNA using the NEBNext rRNA Depletion kit (NEB, E6310).

RNA-seq libraries were then constructed using 10 ng of rRNA-depleted total RNA with Universal Plus mRNA-seq kit (TECAN, 0520-A01) following the kit manual. 2 × 75 bp, 2 × 100 bp, and 2 × 150 bp paired-end sequencing was run for all libraries on the Illumina platforms. Additionally, for tissues harvested at BCM, RNA-seq was performed as described previously ^24,25^ at one of two sites: The MD Anderson Science Park Next Generation Sequencing Core facility using the Illumina TruSeq Stranded Total RNA Protocol and the HiSeq 3000 SBS platform or The University of Houston Core facility using the QIAseq Stranded Total RNA Library Kit and the NextSeq 500 platform.

### ChIP-seq library construction

ChIP-seq was performed as described previously ^24^. Briefly, chromatin was cross-linked with formaldehyde, the reaction was stopped with glycine, and the samples were sonicated with a Bioruptor to obtain fragment sizes of 100–300 bp for ChIP-seq. ChIP-validated antibodies used were directed against H3K4me3 (Active Motif #39915), H3K4me1 (Abcam ab8895), H3K27ac (Abcam ab4729), H3K27me3 (Active Motif #61017), and H3K9me3 (Active Motif #61013). Immunoprecipitated DNA was recovered and purified using the QIAquick PCR Purification kit (Qiagen), and DNA concentration was measured using a NanoDrop spectrophotometer. ChIP-sequencing was performed by the University of Houston Core facility. Sequencing libraries were prepared using the QIAseq FX DNA library kit or the QIAseq Ultralow Input Library Kit and the NextSeq 500 platform.

### RNA-Seq Analysis

Paired-end reads were trimmed using TrimGalore (https://doi.org/10.5281/zenodo.7598955) and mapped using HISAT2 ^48^ to the mouse genome build mm10, then gene expression data was quantified using featureCounts ^49^ and the GENCODE gene model ^50^. Differentially expressed genes (DEGs) were identified using the R package EdgeR ^51^, with significance achieved for a false discovery rate (FDR) adjusted p-value lower than 0.05 and a fold change exceeding 1.5x. Principal component analysis (PCA), volcano plots, and heatmaps visualizations were generated using the R statistical system. UpSet plots were generated using the R ComplexHeatmap package ^52^. Cell type deconvolution was performed using the MuSiC R package ^53^ using a single-cell liver reference ^32^.

Over-representation analysis (ORA) was performed using DEGs to detect enrichment of gene sets corresponding to pathways and biological processes using publicly available gene signatures. Following the Molecular Signature Database methodology (MSigDB), a hypergeometric test was used to assess the enrichment, with significance achieved at FDR-adjusted p-value < 0.05. Gene Set Enrichment Analysis (GSEA) was performed using the GSEA package, with significance achieved at FDR<0.25. We performed ORA and GSEA against the Gene Ontology Biological Process (GOBP), HALLMARK, KEGG, and REACTOME gene set compendia as compiled by MSigDB v7.5.1 ^54^, and against liver cell type gene signatures based on a single-cell RNA-Seq liver reference ^32^.

Networks based on enriched pathways in distinct comparisons were derived using a Python script, with node sizes equal to the overall number of significantly enriched pathways, and edge size equal to the number of commonly enriched pathways between two different comparisons. The Cytoscape platform was used for network visualization ^55^. To determine the significance of overlap of enriched GSEA pathways, we used permutation testing with 10,000 random permutations, matching the number of significant pathways in each comparison. The results of the permutations were fitted to a normal distribution, then the final p-value was determined using the R statistical system.

### ChIP-Seq Analysis

Data quality was assessed with FastQC, then mapped using BOWTIE2 ^56^ to the mouse genome build mm10; enriched regions (peaks) were called using MACS2 ^56^. Differentially expressed peaks (DEPs) were determined using diffReps ^57^, with significance achieved at FDR<0.05 and fold change exceeding 1.5x. ChIP-Seq data was visualized using the Circos platform ^58^, along with genome-wide maps using BEDTools and the Integrative Genomics Viewer (IGV; version 2.17.4). Heatmaps of ChIP-Seq signal for differentially enriched peaks (DEPs) were generated using the Deeptools software ^59^.

Quality control for ChIP-Seq was determined using the synthetic Jensen-Shannon distance for both active and repressive marks, which is based on information theory as the distance from an ideal input sample ^60^. We report the above metric for the ChIP-Seq data by aggregating samples for each histone modification and for ENCODE mouse liver ChIP-seq data (encodeproject.org) using the same marks. ChIP-seq QC data are provided in Supplemental Table 12.

Genes associated with DEPs within 3kb from transcription start sites (TSS) were determined using BEDTools ^61^ software. Genes associated with DEPs at mouse enhancers were determined using BEDTools and the Enhancer Atlas database (EnhAtl) ^27^. EnhAtl provides enhancer-gene chromatin loops; since we are assessing regulation across a mixture of tissues, we first used BEDTools to merge enhancers from EnhAtl, thus generating enhancer anchors (Supplemental Table 4); next, we used an inhouse Python code to determine enhancers that overlap with DEPs via BEDTools and then associated affected enhancer anchors with their target genes.

### Signature Based Analysis Using Human Liver Transcriptomes

We used a collection of human liver transcriptomes compiled by the GTEx consortium ^62^. For each gene signature, we used Biomart to convert mouse gene symbols to human homologs. Each gene was converted to z-score across all samples. A signature score for a sample was computed by adding the z-scores of increased DEGs and subtracting the z-scores of decreased DEGs. We computed signature correlation using the Pearson Correlation Coefficient, with significance achieved for p<0.05. In addition to the signatures from our study, we used a published signature of mouse liver aging (4mo to 28mo) from White *et al*, comprised of 105 genes ^63^.

We integrated these signatures with datasets from HCC samples in the Cancer Genome Atlas (TCGA) LIHC ^64^, GSE54238 ^65^, GSE6764 ^66^, and GSE14520 ^67^; NASH samples in GSE83452 ^68^ and GSE89632 ^69^; and NAFLD samples in GSE49541 ^70^ and GSE151158 ^71^. For each cohort, a gene signature activity score was derived by scaling every gene by converting it to a z-score and computing the summed z-score for all genes ^72,73^. Association of signature activity scores with liver disease was assigned by encoding the disease states with numerical values in order of severity, then computing a Pearson correlation coefficient, with significance achieved for p<0.05.

## Supplementary Material

This is a list of supplementary files associated with this preprint. Click to download.

SuppTable1.xlsx

SuppTable2.xlsx

SuppTable3.xlsx

SuppTable4.xlsx

SuppTable5.xlsx

SuppTable6.xlsx

SuppTable7.xlsx

SuppTable8.xlsx

SuppTable9.xlsx

SuppTable10.xlsx

SuppTable11.xlsx

SuppTable12.xlsx

Supplementalfigures.docx

Additionalinformation.docx

Tables.docx

## Data Availability

RNA-seq data and ChIP-seq data can be accessed at the TaRGET II data portal (https://targetepigenomics.org/) and an accompanying ToxiTaRGET database (http://toxitarget.com/) as well as the Gene Expression Omnibus repository (GSE146508). Additional samples were deposited to the Gene Expression Omnibus repository (accession numbers GSE272929, GSE289162, GSE272927). Custom codes are available at the Zenodo repository 10.5281/zenodo.15086523.
